# Prevalence and Associated Risk Factors of Hypertension: A Cross-Sectional Study in Urban Varanasi

**DOI:** 10.1155/2017/5491838

**Published:** 2017-12-03

**Authors:** Shikha Singh, Ravi Shankar, Gyan Prakash Singh

**Affiliations:** ^1^Department of Community Medicine, Institute of Medical Sciences, Banaras Hindu University, Varanasi 221005, India; ^2^Division of Biostatistics, Department of Community Medicine, Institute of Medical Sciences, Banaras Hindu University, Varanasi 221005, India

## Abstract

Hypertension is a major public health problem and important area of research due to its high prevalence and being major risk factor for cardiovascular diseases and other complications.* Objectives*. (1) To assess the prevalence of hypertension and its associated factors and (2) to estimate awareness, treatment, and adequacy of control of hypertension among study subjects.* Methods and Materials*. A community based cross-sectional study with multistage sampling design was conducted among urban population of Varanasi. A modified WHO STEPS interview schedule on 640 study subjects aged 25–64 years was used.* Results*. The prevalence of hypertension was 32.9% (male: 40.9%, female: 26.0%). Mean systolic and diastolic BP were 124.25 ± 15.05 mmHg and 83.45 ± 9.49 mmHg, respectively. Higher odds of being hypertensive were found in male subjects, eldest age group, married subjects, subjects of upper socioeconomic status, illiterate subjects, and retired subjects. Tobacco and alcohol consumption, overweight, obesity, and abdominal obesity were also associated with hypertension. Out of the total hypertensive 211 subjects, only 81 (38.4%) were aware about their hypertension status; out of those, 57 (70.4%) were seeking treatment and 20 (35.08%) had their blood pressure adequately controlled.* Conclusion*. Around one-third of the subjects were hypertensive and half of the study subjects were prehypertensive in this area. The awareness, treatment, and control of high blood pressure were also very low.

## 1. Introduction

Hypertension is a major public health problem due to its high prevalence all around the globe [1–4]. Around 7.5 million deaths or 12.8% of the total of all annual deaths worldwide occur due to high blood pressure [5]. It is predicted to be increased to 1.56 billion adults with hypertension in 2025 [6].

Raised blood pressure is a major risk factor for chronic heart disease, stroke, and coronary heart disease. Elevated BP is positively correlated to the risk of stroke and coronary heart disease. Other than coronary heart disease and stroke, its complications include heart failure, peripheral vascular disease, renal impairment, retinal hemorrhage, and visual impairment [5].

Hypertension (or HTN) or high blood pressure is defined as abnormally high arterial blood pressure. According to the Joint National Committee 7 (JNC7), normal blood pressure is a systolic BP < 120 mmHg and diastolic BP < 80 mm Hg. Hypertension is defined as systolic BP level of ≥140 mmHg and/or diastolic BP level ≥ 90 mmHg. The grey area falling between 120–139 mmHg systolic BP and 80–89 mmHg diastolic BP is defined as “prehypertension” [7, 8]. Although prehypertension is not a medical condition in itself, prehypertensive subjects are at more risk of developing HTN [1].

It is a silent killer as very rarely any symptom can be seen in its early stages until a severe medical crisis takes place like heart attack, stroke, or chronic kidney disease [8–10]. Since people are unaware of excessive blood pressure, it is only through measurements that detection can be done. Although majority of patients with hypertension remain asymptomatic, some people with HTN report headaches, lightheadedness, vertigo, altered vision, or fainting episode [11].

There are several factors predisposing to hypertension. These factors vary from country to country and even there is difference between urban and rural regions of the same place [12]. Realizing the effect of urbanization on our collective health, World Health Organization has chosen “Urbanization and Health” as the theme for World Health Day 2010 [13]. Urbanization is considered a determinant of health and one of the key drivers of noncommunicable diseases (NCDs), especially in low- and middle-income countries (LMICs) [14]. Urban people are more at risk of these diseases as compared to their rural counterparts. As per the findings of National Family Health Survey (NFHS-4), the prevalence of hypertension, obesity, and blood glucose in urban area of Uttar Pradesh was 10.5%, 23.9, and 9.9%, respectively. However, the prevalence of the same phenomenon was 8.3%, 10.8%, and 8.2%, respectively in rural area [15]. It is clear that all the parameters are having higher prevalence in urban area as compared to rural area. Rapid urbanization, increasing elderly population, mechanization, sedentary life, and dietary changes act together as a web of risk factors which entangles people in it and leads to several chronic diseases. In order to take effective prevention measures, identification of the risk factors is an essential prerequisite. This study intends to generate information on prevalence of hypertension and their associated risk factors in urban area of Varanasi. In addition, it will also look into the awareness and control of hypertension among the study subjects.

## 2. Materials and Methods

### 2.1. Study Area

Varanasi is an Indian city on the bank of Ganges in Uttar Pradesh. It has total population of 3676841 as per Census 2011. As per Census 2011, out of total population, 52% people live in urban areas, while 48% live in the rural areas. There are 90 Census enumeration wards in Varanasi district. Out of these 90 wards, 5 wards were selected by using simple random sampling.

### 2.2. Study Design and Sample Size

A community based cross-sectional study was carried out among the people aged 25 to 64 years living in the selected study area. The sample size for the present study was calculated by taking most probable prevalence of hypertension as 50% and permissible error as 5% with 95% confidence interval. Fixing the permissible error as 50%, the minimum sample size was calculated as *n* = 384. Since sampling procedure was multistage, hence considering the design effect, the sample size was further increased by one and half times. Considering the nonresponse rate of 10% the final sample size in study was fixed as 640. In the present study, a prior written informed consent was also taken from the participants. Prior written informed consent was taken by the participants.

### 2.3. Sampling Methodology

A multistage sampling was used for this study. There were three stages and for each stage different sampling design was used.

At first, out of these 90 wards, 5 wards were selected by using simple random sampling. At second stage, from each selected ward the households were further selected by using systematic random sampling and probability proportional to size was done. At the third stage, one member of target age group was interviewed from selected household. If the selected family has more than one available eligible person then one was chosen randomly by using lottery method. In case of nonavailability of eligible person in a selected household, at the time of survey, the adjacent household was selected.

### 2.4. Selection of Study Subjects

#### 2.4.1. Inclusion Criteria

Individuals aged 25–64 years in the selected study area who gave consent for participation were considered.

#### 2.4.2. Exclusion Criteria

Individuals who are unable to give response due to serious physical or mental illness and with whom anthropometry measurements cannot be performed were excluded from the study.

### 2.5. Tools of the Study

Interview schedule [modified and pretested WHO stepwise approach to chronic disease risk factor surveillance (STEPS)], Libra weighing machine, steel anthropometry rod, measuring tape, and Omron BP Machine were used.

### 2.6. Techniques of the Study

In all study participants, a structured and pretested interview schedule was administered to obtain data on sociodemographic parameters.

#### 2.6.1. Blood Pressure Measurement

Blood pressure was measured two times on the right arm of the selected subject using automatic electronic device (OMRON HEM-7261). The average of two readings was used.

#### 2.6.2. Anthropometric Measurements

All the anthropometric measurements were done by the following standardized technique. Weight was measured by Libra weighing machine having an accuracy of 0.1 kg and height was measured by using a steel anthropometry rod with accuracy of 0.1 cm using standard techniques. Body Mass Index was calculated using the following formula: BMI = weight (kg)/height (mt)^2^. Based on BMI obtained, the subjects were classified into different categories according to the WHO global classification [16]. Waist circumference (in cm) was measured using a nonstretchable measuring tape. Waist circumference was measured at the smallest horizontal girth between the costal margins and the iliac crest at the end of expiration. Hip circumference (in cm) was measured at the broadest part of the hips by using nonstretchable measuring tape. Waist-to-hip circumference (WHR) was calculated by dividing waist circumference by hip circumference [17].

### 2.7. Ethical Consideration

Ethical approval was obtained from the Institute Ethical Committee of the Institute of Medical Sciences, Banaras Hindu University Varanasi. Prior written consent was taken from the subjects who volunteered to participate in the study. Identified hypertensive subjects were referred to the nearby clinic for treatment.

### 2.8. Definitions Used

(i) Joint National Committee on Prevention, Detection, Evaluation, and Treatment of High Blood Pressure (JNC 7) classification was used for hypertension [8].
(ii) Hypertension is defined as systolic BP level of ≥140 mmHg and/or diastolic BP level of ≥90 mmHg or being previously diagnosed as hypertensive by any health professional. The area falling between 120–139 mmHg systolic BP and 80–89 mmHg diastolic BP is defined as “prehypertension” [8].
(iii) Isolated diastolic hypertension (IDH) having a systolic blood pressure ≤ 140 mmHg and diastolic blood pressure ≥ 90 mmHg and isolated systolic hypertension (ISH) having a systolic blood pressure ≥ 140 mmHg and diastolic blood pressure < 90 mmHg was used to diagnose IDH and ISH, respectively.
(iv) Awareness was defined as history of hypertension based on diagnosis by a healthcare provider. Treatment was defined as taking any medication or other treatment for hypertension in the last two weeks prior to the survey and control was defined as blood pressure < 140 and <90 mmHg in subjects who were taking medications
(v) WHO International BMI classification: BMI < 18.5 was classified as* “underweight”;* <16.00,* “severe thinness”;* 16.00–16.99,* “moderate thinness”;* 17.00–18.49,* “mild thinness”;* 18.50–24.99,* “normal range”;* BMI ≥ 25.00,* “overweight”*; 25.0–29.99,* “preobese”*; ≥30.00,* “obese”*; 30.00–34.99,* “obese class I”*; 35.00–39.99,* “obese class II”;* and >40.00,* “obese class III.”*
(vi) Socioeconomic class categorization was done by using modified BG Prasad classification according to all India consumer price index (AICPI) for the month of January 2014 [18] (Table 1).
  (1) $$\begin{array}{c} \text{Multiplication Factor}=\frac{\text{Value of price index}\times 4.93}{100}. \end{array}$$
(vii) Current daily smokers are defined as those who were currently smoking cigarettes, bidis, or hookah daily. Current daily smokeless tobacco users are defined as those who were currently using chewable tobacco products, gutka, naswar, khaini, or zarda paan daily. Current alcohol drinkers are defined as those who reported to consuming alcohol within the past one year [17].
(viii) Physical activity was measured in three domains that is activity at work, to and from places, and recreational activities as well as time spent sitting. The interview schedule also covered type of activity (vigorous and moderate) at work and for recreational activities. Information was also collected on the number of days in a week spent on different activities and time spent in a day for each activity was also recorded [17]. Those who were not active in any domain were defined as “inactive,” those who were vigorously active in any category were defined as “vigorously active,” and the rest were “moderately active.”

### 2.9. Data Processing

The information obtained from the survey was entered into a database developed for the study, using SPSS 16.0 program. Descriptive statistics (mean and standard deviation) were calculated for continuous variables and frequencies and percentages were calculated to summarize qualitative data. Other statistical tests like chi-square test and ANOVA were applied. Logistic regression was applied to identify the risk factors for hypertension. A significance level of 0.05 was used.

## 3. Results

A total of 640 study subjects were interviewed for the survey. Out of these, 301 (47%) were male subjects and 339 (53%) were female. The median age (±SD) of the study subjects was 39.0 (±11.9) years and for male and female it was 40.0 (±11.9) years and 38 (±11.8) years, respectively. Regarding religion and caste of the study subjects, around 96% subjects were Hindu and majority of the subjects were in general category, respectively. Majority of the study subjects were married and one-third of the subjects belonged to the upper socioeconomic class. Mean (±SD) BMI of the study subjects was 24.11 ± 3.94 kg/m^2^; for men it was 23.78 ± 3.95 kg/m^2^ and for women it was 24.41 ± 3.92 kg/m^2^. According to Body Mass Index (BMI), more than one-third of the study subjects were either overweight or obese. With regard to abdominal obesity as measured by waist circumference, 40% subjects were at risk (Table 2).

Table 3 depicts the mean values of systolic and diastolic BP according to age and gender. The mean systolic and diastolic BP of all the study subjects were 124.2 ± 15.0 mmHg and 83.4 ± 9.5 mmHg, respectively. In men, the highest mean systolic BP and mean diastolic BP were among the eldest age group and preceding eldest age group (45–54 years), respectively, while in female the highest mean value of systolic and diastolic BP both were among the 45–54-year age group. With regard to systolic BP, there was significant difference among all the age groups among both male and female study subjects and the same was with diastolic BP as well. The prevalence of isolated systolic BP was found to be 10.6% [95% CI: (8.27–13.37)] and isolated diastolic BP was 19.7% [95% CI: 16.6–23.18]. The proportion was higher in male (14.8%) as compared to female (6.8%). Among both groups (male and female), prevalence was higher among the eldest age group. The prevalence of isolated diastolic BP was higher among male subjects (28.1) against female subjects (12.2%). It was the highest among the second oldest age group among male and oldest age group in female subjects. With regard to systolic BP, age was associated with hypertension status among both genders, whereas diastolic BP was associated with age in male subjects only. There was no association between age and diastolic BP in female subjects.

The overall prevalence of hypertension was 32.96% [95% CI: (29.4–36.7)]. The sex specific prevalence was 40.9% [95% CI: 35.5–46.5] for male and 26.0% [95% CI: 21.6–30.9] for female. Prehypertension was prevalent in 45.9% [95% CI: (40.3–51.5)] of men and 38.05% [95% CI: (33.0–43.2)] of women. In men, hypertension was significantly associated with age but in women, age does not have any effect on their hypertension status. History of hypertension or the prediagnosed cases of hypertension was more among female (6.7%) as compared to male (5.9%) subjects (Table 4).

Table 5 shows the associated factors of prehypertension and hypertension. Gender, age, marital status, occupation, education status, tobacco use, and physical activity were significantly associated with the hypertension status of the study subjects (*p* < 0.05). Both the rate of prehypertension and hypertension were higher among male. Hypertension was more prevalent in the 45–54 years, while prehypertension was more in the 35–44-year age group. Being married and government servant were found to be risk factors for both hypertension and prehypertension. Hypertension was found to be more among illiterate subjects, and with regard to prehypertension, primary educated subjects suffered more. Study subjects from lower and upper socioeconomic status were almost equal victims of hypertension. Tobacco use and alcohol use were found to be risk factors for being hypertensive in the study subjects. Although alcohol use was not significantly associated with hypertension status but rate of hypertension was higher among the alcohol users.

The binary logistic regression analysis showed that odds of being hypertensive were higher among the male subjects (OR: 1.97), eldest age group (OR: 6.49), married subjects (OR: 2.34), uneducated subjects (OR: 1.17), retired subjects (OR: 3.66), and those who were from upper socioeconomic status (1.31). With regard to anthropometric risk factors, being overweight (OR: 1.99), being obese (OR: 3.57), and having abdominal obesity (OR: 1.73) had higher odds of hypertension. Tobacco use (OR: 1.86), alcohol use (OR: 1.55), and nonvegetarian diet (OR: 1.10) also had higher odds of being hypertensive. Gender, age, marital status, occupation, BMI, abdominal obesity, and tobacco use were significantly associated with hypertension. Education, socioeconomic status, and alcohol use were not statistically associated with hypertension. Being female, younger in age, unmarried, highly educated, and staying away from any kind of addiction could serve as protective factors against hypertension (Table 6).

**Figure 1:**
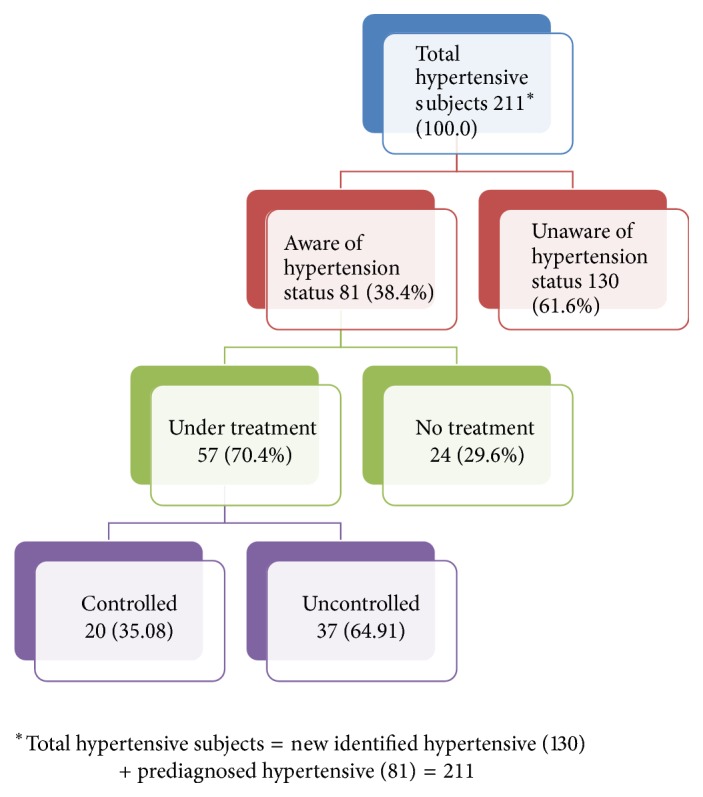
Flow diagram showing awareness, treatment, and adequacy of control of hypertension among study subjects.

Out of the total subjects with hypertension, around one-third of the subjects were aware of their condition. Out of those who were aware, 70% were seeking treatment. Only a third of the treated subjects with hypertension had their blood pressure adequately controlled (Figure 1). Females were marginally more aware of their hypertensive status as compared to male counterparts (see Table 4, history of hypertension). As age, education status, and socioeconomic status were advancing, the awareness of hypertensive status among study subjects was also increasing (not shown in the table).

## 4. Discussion

India is a developing country and like other developing countries, it is going through a rapid demographic and epidemiological transition. In all such transitions, nutrition is the key ingredient and plays prime role. This cross-sectional community based study identified a high prevalence of prehypertension and hypertension in urban areas of Varanasi, which was 41.7%   and 32.96%, respectively. Only a quarter of subjects were in the normal category, which highlights the escalating burden of this silent killer.

The prevalence of hypertension in the present study (32.96%) was higher in comparison with the prevalence reported in other studies [4, 6, 10, 19–22]. Few studies reported the results in line with the present study [23, 24]. According to World Health Organization (2015), the overall prevalence of hypertension in India was 23.5% and gender specific prevalence was 24.2%  and 22.7% among the men and women, respectively [25].

The prevalence of prehypertension in the present study was 41.7% (male: 45.9% and female: 38.05%). The prevalence estimated in the present study was much higher than that estimated by Nellore (22.3%) [10] and Bihar (37.95%) [20]. The difference of prevalence observed between the present study and other studies with respect to hypertension and prehypertension could be due to social and cultural differences, dietary and lifestyle factors, and also the age span as well as the research methodology used.

Men exhibit higher prevalence of hypertension and prehypertension than their female counterparts (M: 40.9%  and F: 26.0%) and (M: 45.9%  and F: 38.05%), respectively. Similarly, various studies came out with the higher percentage of hypertension in men than women [20, 22, 23, 26–29]. One of the possible explanations for this gender disparity in hypertension prevalence could be partially due to biological sex difference and partially due to behavioral risk factors like smoking, alcohol consumption, or physical activity. We speculate that absentia from alcohol and smoking might be few of those protective factors against hypertension in women. Other than that, women are more interested in health care services utilization and also more frequently report their poor health and therefore they are more likely to have better health [6, 30].

Age was found to be an important risk factor for hypertension. As the age was advancing so did the prevalence of hypertension among both the sexes. Similar findings were reported by few other studies also where advancing age was positively related to hypertension [1, 6, 19, 21, 22, 26, 31, 32]. With increasing age, the aorta and arteries walls will be stiffened and this contributes to the high prevalence of hypertension in older age groups [4].

In the present study, marital status, education, occupation, socioeconomic status, BMI, abdominal obesity, tobacco use, alcohol use, and physical activity were significantly associated with the hypertension.

Low literacy level and being too rich were associated with hypertension. The higher education level was negatively correlated to hypertension in the present study. These studies also supported this finding [6, 12, 33]. We speculate that it could be due to the reason that higher education imparts better knowledge and information about hypertension and subsequently those people with higher education had a healthier lifestyle.

Table 5 revealed that education was significantly associated with hypertension (*χ*^2^ = 17.049, df = 6 and *p* value = 0.009); however, when adjusted effect of education on hypertension was observed by logistic regression, then no statistical association was observed. Though some studies had shown a significant association of these two variables [6, 12], in the study performed in the state of Kerala [33], insignificant association between education and hypertension was observed. We speculate that this insignificant association could be due to very few subjects in the illiterate and less educated category. There are so many studies which do not refute the finding of the present study that higher socioeconomic status is a risk for hypertension [3, 22, 24, 32, 34–36]. We assume that better socioeconomic status imparts people with more purchasing power on fast and convenience foods and less physical activity which are already proven to be contributing risk factors for overweight and obesity that subsequently linked to hypertension.

The different anthropometric measurements like BMI, waist circumference, and hip circumference were taken into account to measure overweight, obesity, and central or abdominal obesity. This study showed that overweight and obesity measured by both BMI and waist circumference were major modifiable risk factors to develop hypertension. Overweight subjects had twofold risk of being hypertensive and obese had more than threefold risk for the same in comparison to underweight subjects in this study. There was positive relation observed between increasing BMI and increasing rate of hypertension, which was consistent with other studies [1–4, 6, 12, 20, 21, 23, 32, 36–39]. South Asians have tendency of developing centralized obesity without developing generalized obesity and because of this waist circumference and waist-hip ratio are better measures of body fat [40]. Abdominal obesity (OR: 1.73) also found to be positively linked to high blood pressure in the present study. Various epidemiological and pathophysiological mechanisms explained the link between obesity and hypertension. One of the probable reasons behind this positive relation between obesity and hypertension could be that increased weight increases cardiac output and increased peripheral resistance of arterioles. Other than that, urbanization is also a cause of changes in dietary habits and reduced physical activity which leads to obesity and subsequently results in hypertension [4].

Interestingly, we have found inverse association between physical activity and hypertension. Hypertension was more among physically active subjects as compared to inactive subjects (OR: 0.63) but no statistically significant difference was found. Similar result was reported by other study conducted in Turkey [1]. The exact reason behind this is unknown and yet to be explored. We speculate that they had started physical activity probably under medical advice after being diagnosed for hypertension or other risk factors like overweight or obesity.

As per WHO report, alcohol consumption was the third largest risk factor in the developed countries and tobacco use was being the second major cause of death worldwide [17]. This study indicated the positive association between alcohol and tobacco use and hypertension. Hypertension was more prevalent in tobacco users (OR: 1.86) and alcohol users (OR: 1.55) as compared to nonusers. This finding is supported by other studies also [19, 23, 32, 36]. But there are several other studies with contradictory findings [1, 21, 41]. Although not statistically significant, odds of being hypertensive were more among nonvegetarian (OR: 1.10) subjects, while vegetarian diet was proved to be protective against hypertension in this study. Several other studies reported the same result [3, 19, 33, 34]. A study conducted in Bihar [20] refutes this finding and reported that vegetarian diet was positively associated with hypertension.

A recent review study revealed that hypertension awareness rate has been doubled from less than 30% in 1980s to around 60% in present among urban populations and less than 10% in 1980s to 35–40% presently among rural population. However, the treatment and control status is still low at around 30% in urban and 20% in rural areas [42]. Rate of awareness, treatment, and control in the present study was observed as 38.4%, 70.4%, and 35.08%, respectively. Previous study conducted in rural Varanasi reported hypertension awareness, treatment, and control (26.5%, 55.6%, and 40%), respectively [7].

## 5. Conclusion & Recommendation

From the results of this study, it can be concluded that the prevalence of both prehypertension and hypertension is very high in urban Varanasi. This makes the people of this area vulnerable to several chronic diseases and other unbearable health consequences. Specifically men are at more risk of being hypertensive than female. Increasing age is proved to be an independent risk factor for hypertension. Programs are needed to improve the surveillance systems and implementation of community based screening programs for early detection of hypertension is also needed. As the awareness of the hypertension status among hypertensive cases was very poor, improving health literacy to increase the awareness of hypertension is also the need of the hour. Interventions like weight management, increased physical activity, increased fruits and vegetables consumption, and reduction in tobacco and alcohol use are required and recommended.

## Figures and Tables

**Table 1:** Revised modified BG Prasad socioeconomic classification scale, January 2014.

Socioeconomic class	Per capita monthly income	
	1961 (base year)	Revised income categories 2014
Upper class	100 and above	5357 and above
Upper middle class	50–99	2652–5356
Middle class	30–49	1570–2651
Lower middle class	15–29	812–1569
Lower class	<15	<811

**Table 2:** Background characteristics of the study subjects (*N* 640).

Variables	*N* (640)	Proportion%
Age group		
25–34	204	31.9
35–44	179	28.0
45–54	133	20.8
55–64	124	19.4
Sex		
Male	301	47.0
Female	339	53.0
Caste		
Gen	313	48.9
OBC	305	47.7
SC/ST	22	3.4
Religion		
Hindu	619	96.7
Non-Hindu	21	3.3
Education		
Illiterate	64	10.0
Primary	85	13.3
Secondary	182	28.4
Graduate & above	309	48.3
Marital status		
Unmarried	60	9.4
Married	545	85.2
Others	35	5.5
Occupation		
Government	86	13.4
Private	155	24.2
Self-employed	109	17.0
Retired	27	4.2
Homemaker	229	35.8
Others	34	5.3
Family type		
Nuclear	311	48.6
Joint	329	51.4
Number of family members		
1–5	401	62.7
≥6	239	37.3
Socioeconomic status		
Lower class	41	6.4
Lower middle class	105	16.4
Middle class	120	18.8
Upper middle class	140	21.9
Upper class	234	36.6
BMI (kg/m^2^)		
Overweight	186	29.1
Obese	59	9.2
Waist circumference (cm)		
Abdominal obesity	258	40.3

**Table 3:** Mean systolic and diastolic blood pressure (mm hg) and prevalence (%) of isolated systolic hypertensive and isolated diastolic hypertensive by age and gender.

Age groups (years)	*N* 640	Systolic BP (mean ± SD)			Diastolic BP (mean ± SD)		
		Male	Female	Total	Male	Female	Total
25–34	*204*	122.17 ± 9.54	114.81 ± 9.99	117.84 ± 10.44	82.92 ± 9.0	78.97 ± 7.46	80.59 ± 8.34
35–44	*179*	124.10 ± 10.77	121.71 ± 15.13	122.90 ± 13.07	85.70 ± 7.66	81.71 ± 9.30	83.75 ± 8.68
45–54	*133*	132.36 ± 13.21	127.16 ± 18.04	129.66 ± 16.05	*89.23 ± 8.16*	*83.28 ± 10.22*	*86.14 ± 9.72*
55–64	*124*	*134.66 ± 19.53*	*127.27 ± 15.74*	*130.97 ± 18.05*	86.42 ± 12.15	83.24 ± 9.32	84.83 ± 10.90
Total	*640*	127.49 ± 14.19	121.39 ± 15.26	124.25 ± 15.05	85.82 ± 9.43	81.34 ± 9.05	83.45 ± 9.49
*Test of significance*		*F = 15.396*	*F = 15.611*	*F = 30.466*	*F = 5.801*	*F = 4.921*	*F = 11.174*
		*df = 3*	*df = 3*	*df = 3*	*df = 3*	*df = 3*	*df = 3*
		*p = 0.001*	*p = 0.001*	*p = 0.001*	*p = 0.001*	*p = 0.002*	*p = 0.001*

Age groups (years)	*N* 559	Isolated systolic HTN (*N* = 59)^#^			Isolated diastolic HTN (*N* = 110)^#^		
		Male	Female	Total	Male	Female	Total

25–34	202	3 (3.6)	1 (0.8)	4 (6.8)	14 (16.7)	10 (8.5)	24 (21.8)
35–44	166	11 (12.9)	7 (8.6)	18 (30.5)	25 (29.4)	10 (12.3)	35 (31.8)
45–54	101	9 (18.4)	6 (11.5)	15 (25.4)	22 (44.9)	8 (15.4)	30 (27.3)
55–64	90	16 (35.6)	6 (13.3)	22 (37.3)	13 (28.9)	8 (17.8)	21 (19.1)
Total		*39 (14.8)*	*20 (6.8)*	*59 (10.6)*	*74 (28.1)*	*36 (12.2)*	*110 (19.7)*
*Test of significance*		*χ* ^*2*^ *= 24.461*	*χ* ^*2*^ *= 11.974*	*χ* ^*2*^ *= 36.114*	*χ* ^*2*^ *= 12.355*	*χ* ^*2*^ *= 3.338*	*χ* ^*2*^ *= 15.160*
		*df = 3*	*df = 3*	*df = 3*	*df = 3*	*df = 3*	*df = 3*
		*p = 0.001*	*p = 0.007*	*p = 0.001*	*p = 0.006*	*p = 0.342*	*p = 0.002*

^#^Excluding known hypertensive.

**Table 4:** Prevalence of hypertension and prehypertension by gender and age groups among the study subjects (*N* 640).

Category	*n*	Age groups (years)				Test of significance
		25–34	35–44	45–54	55–64	
*Men (n 263)*		*84*	*85*	*49*	*45*	*χ* ^*2*^ * = 22.297* *df = 9* *p = 0.008*
Normal	*40*	18 (45.0)	10 (25.0)	3 (7.5)	9 (22.5)	
Prehypertension	*138*	51 (37.0)	48 (34.8)	21 (15.2)	18 (13.0)	
HTN stage 1	*69*	12 (17.4)	23 (33.3)	19 (27.5)	15 (21.7)	
HTN stage 2	*16*	3 (18.8)	4 (25.0)	6 (37.5)	3 (18.8)	
History of HTN	*38*	0 (0.0)	6 (15.8)	15 (39.5)	17 (44.7)	
*Women (n 296)*		*118*	*81*	*52*	*45*	*χ* ^*2*^ * = 13.983* *df = 9* *p = 0.123*
Normal	*122*	59 (48.4)	33 (27.0)	17 (13.9)	13 (10.7)	
Prehypertension	*129*	48 (37.2)	35 (27.1)	25 (19.4)	21 (16.3)	
HTN stage 1	*37*	10 (27.0)	10 (27.0)	7 (18.9)	10 (27.0)	
HTN stage 2	*8*	1 (12.5)	3 (37.5)	3 (37.5)	1 (12.5)	
History of HTN	*43*	2 (2.5)	13 (16.0)	32 (39.5)	34 (42.0)	

**Table 5:** Prevalence of prehypertension and hypertension^*∗*^ according to sociodemographic characteristics and behavioral risk factors.

Variables	Total *N* 559	Normal	Prehypertension	Hypertension	Test of significance
Sex					
Male	*263*	40 (15.2)	138 (52.5)	85 (32.3)	*p = 0.001* *df = 2* *χ*^*2*^* = 52.352*
Female	*296*	122 (41.2)	129 (43.6)	45 (15.2)	
Age					
25–34	*202*	77 (38.1)	99 (49.0)	26 (12.9)	*p = 0.001* *df = 6* *χ*^*2*^* = 28.822*
35–44	*166*	43 (25.9)	83 (50.0)	40 (24.1)	
45–54	*101*	20 (19.8)	46 (45.5)	35 (34.7)	
55–64	*90*	22 (24.4)	39 (43.3)	29 (32.2)	
Caste					
Gen	*263*	62 (23.6)	140 (53.2)	61 (23.2)	*p = 0.087* *df = 4* *χ*^*2*^* = 8.12*
OBC	*275*	93 (33.8)	118 (42.9)	64 (23.3)	
SC/ST	*21*	7 (33.3)	9 (42.9)	5 (23.8)	
Marital status					
Unmarried	*58*	22 (37.9)	27 (46.6)	9 (15.5)	*p = 0.033* *Df = 4* *χ*^*2*^* = 10.501*
Married	*473*	126 (26.6)	231 (48.8)	116 (24.5)	
Others	*28*	14 (50.0)	9 (32.1)	5 (17.9)	
Occupation					
Government	*75*	4 (5.3)	40 (53.3)	31 (41.3)	*p = 0.001* *df = 10* *χ*^*2*^* = 51.488*
Private	*142*	36 (25.4)	75 (52.8)	31 (21.8)	
Self-employed	*100*	27 (27.0)	45 (45.0)	28 (28.0)	
Retired	*15*	4 (26.7)	7 (46.7)	4 (26.7)	
Homemaker	*194*	82 (42.3)	87 (44.8)	25 (12.9)	
Others	*33*	9 (27.3)	13 (39.4)	11 (33.3)	
Education					
Illiterate	*57*	23 (40.3)	18 (31.6)	16 (28.1)	*p = 0.009* *df = 6* *χ*^*2*^* = 17.049*
Primary	*71*	25 (35.2)	34 (47.9)	12 (16.9)	
Secondary	*157*	53 (33.7)	67 (42.7)	37 (23.6)	
Graduate & above	*274*	61 (22.3)	148 (54.0)	65 (23.7)	
Socioeconomic status					
Lower	*38*	11 (28.9)	15 (39.5)	12 (31.6)	*p = 0.001* *df = 8* *χ*^*2*^* = 30.454*
Lower middle	*99*	42 (42.4)	37 (37.4)	20 (20.2)	
Middle	*112*	37 (33.0)	51 (45.5)	24 (21.4)	
Upper middle	*120*	39 (32.5)	64 (53.3)	17 (14.2)	
Upper	*190*	33 (17.4)	100 (52.6)	57 (30.0)	
Tobacco use					
Nonusers	*391*	127 (32.5)	188 (48.1)	76 (19.4)	*χ* ^*2*^ * = 13.685* *df = 2* *p = 0.001*
Users	*168*	35 (20.8)	79 (47.0)	54 (32.1)	
Alcohol use					
Nonusers	*498*	149 (29.9)	240 (48.2)	109 (21.9)	*χ* ^*2*^ * = 5.239* *df = 2* *p = 0.073*
Users	*61*	13 (21.3)	27 (44.3)	21 (34.4)	
Physical activity					
Inactive	*55*	10 (18.2)	36 (65.5)	9 (16.4)	*χ* ^*2*^ * = 7.968* *df = 2* *p = 0.021*
Active	*504*	152 (30.2)	231 (45.8)	121 (24.0)	

^*∗*^Excluding known hypertensive.

**Table 6:** Univariate analysis for the association of hypertension and sociodemographic characteristics, anthropometric measurements, and behavioral risk factors (*N* 640).

Variables	Odds ratio (95% CI)	*p* value
Sex		
Male	1.97 (1.41–2.75)	0.001
Female	1.0 (reference)	
Age groups		
25–34	1.0 (reference)	
35–44	2.64 (1.58–4.41)	0.001
45–54	6.38 (3.77–10.77)	0.001
55–64	6.49 (3.814–11.05)	0.001
Marital status		
Unmarried	1.0 (reference)	
Married	2.34 (1.19–4.61)	0.014
Others	2.32 (0.89–6.04)	0.084
Education		
Graduate & above	1.0 (reference)	
Illiterate	1.17 (0.66–2.06)	0.580
Primary	0.92 (0.54–1.54)	0.756
Secondary	1.08 (0.73–1.59)	0.698
Occupation		
Private	1.0 (reference)	
Government	2.40 (1.39–4.16)	0.002
Self-employed	1.29 (0.76–2.19)	0.336
Retired	3.66 (1.57–8.52)	0.003
Homemaker	0.89 (0.56–1.41)	0.636
Others	1.37 (0.62–3.01)	0.426
Socioeconomic status		
Lower class	1.0 (reference)	
Lower middle class	0.57 (0.26–1.23)	0.156
Middle class	0.63 (0.29–1.33)	0.230
Upper middle class	0.62 (0.29–1.30)	0.208
Upper class	1.31 (0.66–2.61)	0.432
BMI		
Underweight	1.0 (reference)	
Normal	1.27 (0.65–2.47)	80.480
Overweight	1.99 (1.00–3.97)	0.049
Obese	3.57 (1.59–8.00)	0.002
Abdominal obesity		
No	1.0 (reference)	
Yes	1.73 (1.24–2.42)	0.001
Tobacco use		
Nonusers	1.0 (reference)	0.001
Users	1.86 (1.31–2.64)	
Alcohol use		
Nonusers	1.0 (reference)	0.092
Users	1.55 (0.93–2.57)	
Physical activity		
Inactive	0.63 (0.34–1.17)	0.146
Active	1.0 (reference)	
Eating habits		
Vegetarian	1.0 (reference)	
Nonvegetarian	1.10 (0.78–1.54)	0.569
Ovo-vegetarian	0.34 (0.11–1.02)	0.055

## Additional Points

*Limitations of the Study*. This study suffers from few of the following limitations. (i) One of the main limitations of this study was the cross-sectional design of the study which restricts examining causal associations. (ii) The study was only conducted in urban areas. (iii) Stress is a major risk factor for hypertension. It could also be considered in the present study for better results.
